# Predicting response to patients with gastric cancer via a dynamic-aware model with longitudinal liquid biopsy data

**DOI:** 10.1007/s10120-025-01628-4

**Published:** 2025-06-17

**Authors:** Zifan Chen, Jie Zhao, Yanyan Li, Xujiao Feng, Yang Chen, Yilin Li, Xinyu Nan, Huimin Liu, Bin Dong, Lin Shen, Li Zhang

**Affiliations:** 1 Center for Data Science, Peking University, Haidian District, Beijing, 100080 China; 2 Department of Gastrointestinal Oncology, Key Laboratory of Carcinogenesis and Translational Research (Ministry of Education), Peking University Cancer Hospital and Institute, Beijing, China; 3 National Engineering Laboratory for Big Data Analysis and Applications, Peking University, Beijing, China; 4 Guangzhou Medical University, Guangzhou, China; 5 Beijing International Center for Mathematical Research and the New Cornerstone Science Laboratory, Peking University, Beijing, China; 6 Center for Machine Learning Research, Peking University, Beijing, China; 7 Peking University Changsha Institute for Computing and Digital Economy, Changsha, China

**Keywords:** Gastric cancer, Longitudinal liquid biopsy data, Treatment response prediction, Artificial intelligence, Deep learning

## Abstract

**Background:**

Gastric cancer (GC) presents challenges in predicting treatment responses due to its patient-specific heterogeneity. Recently, liquid biopsies have emerged as a valuable data modality, offering essential cellular and molecular insights while facilitating the capture of time-sensitive information. This study aimed to leverage artificial intelligence (AI) technology to analyze longitudinal liquid biopsy data.

**Methods:**

We collected a dataset from longitudinal liquid biopsies of 91 patients at Peking Cancer Hospital, spanning from July 2019 to April 2022. This dataset included 1895 tumor-related cellular images and 1698 tumor marker indices. Subsequently, we introduced the Dynamic-Aware Model (DAM) to predict responses to GC treatment. DAM incorporates dynamic data through AI-engineered components, facilitating an in-depth longitudinal analysis.

**Results:**

Utilizing threefold cross-validation, DAM exhibited superior performance compared to traditional cell-counting methods, achieving an AUC of 0.807 in predicting GC treatment responses. In the test set, DAM maintained stable efficacy with an AUC of 0.802. Besides, DAM showed the capability to accurately predict treatment responses based on early treatment data. Moreover, DAM’s visual analysis of attention mechanisms identified six dynamic visual features related to focus areas, which were strongly associated with treatment-response.

**Conclusions:**

These findings represent a pioneering effort in applying AI technology to interpret longitudinal liquid biopsy data and employ visual analytics in GC. This approach provides a promising pathway toward precise response prediction and personalized treatment strategies for patients with GC.

**Supplementary Information:**

The online version contains supplementary material available at 10.1007/s10120-025-01628-4.

## Background

Gastric cancer (GC), which ranks fifth globally in prevalence, is the third leading cause of cancer-related mortality [1, 2]. The widespread occurrence of GC demands the development of effective treatments and precise predictive models for treatment responses [3, 4]. However, the treatment of GC is complicated by patient heterogeneity, which impedes precise predictions of treatment responses [5, 6]. This heterogeneity, stemming from cellular characteristics and morphologic variations, is crucial for devising patient-specific treatment plans [7, 8]. In response, recent advancements in the analysis of liquid biopsy data, including circulating tumor cells (CTCs), circulating endothelial cells (CECs), and tumor markers, have emerged as powerful tools in cancer treatment management [9, 10]. For instance, previous studies utilizing single-cell sequencing have confirmed that the genomics and transcriptomics of CTCs exhibit significant heterogeneity, with mutation profiles that are partially consistent with, yet distinct from, those of primary and metastatic tumors. This supports the reliability of CTCs as a source for heterogeneity studies [11, 12]. Furthermore, the clonal diversity of CTCs can reveal functional heterogeneity associated with treatment resistance; for example, CTC clusters demonstrate a higher metastatic potential and possess gene expression profiles that differ from those of individual CTCs [13]. The analysis of tumor-related cells and tumor markers in liquid biopsies offers dual advantages: first, it serves as a cellular-level biomarker, providing insights into cellular heterogeneity and its association with treatment response [14–17]; second, liquid biopsies are more convenient than traditional diagnostic methods, such as computed tomography (CT) scans and histopathological examinations [14, 18], facilitating the collection of time-sensitive data essential for understanding a patient’s evolving cellular changes during treatment [15]. Indeed, longitudinal liquid biopsy data is expected to inform personalized treatment strategies that adapt to the changing nature of the disease and the patient’s response. 

Recent advancements in cancer diagnosis and treatment management have largely benefited from the application of cellular biomarkers, including CTCs [19–22], CECs [23, 24], and tumor markers [25, 26]. These developments have greatly enhanced our understanding of cancer, ranging from early detection [23, 27–29] to the prediction of treatment outcomes [20, 21, 24, 30, 31]. However, many previous studies [32–34] have relied on cell-counting-based statistical approaches for outcome assessment. While these methods are useful, they often overlook potential insights that longitudinal liquid biopsy data can provide. In contrast, artificial intelligence (AI), particularly in sequence modeling [35–37] and visual information extraction [38, 39], holds great promise for revolutionizing biomedical data analysis [40–44]. AI’s capacity to process large datasets and decode complex patterns may lead to more precise and personalized assessments of treatment efficacy [45–47]. By integrating AI into GC management enables the development of uniquely tailored treatment strategies based on each patient’s molecular profile, thereby enhancing treatment effectiveness and minimizing invasiveness [48–50]. Although some studies [51–53] have begun to explore the role of dynamic data in cancer treatment management, to our knowledge, none have yet investigated longitudinal liquid biopsies, particularly concerning dynamic tumor-related cellular images and dynamic tumor markers. This highlights the need for more advanced analytical methods in the analysis of longitudinal liquid biopsy data.

In response to these challenges, the primary objective of this study is to introduce an AI-driven framework capable of modeling longitudinal liquid biopsy data for precise treatment-response prediction. We compiled a comprehensive longitudinal dataset from 91 patients with GC treated at Peking Cancer Hospital between July 2019 and April 2022. This dataset includes 1895 aneuploid tumor-related cellular images and 1698 tumor marker indices derived from six different markers, collected during all available follow-up visits. We randomly divided the dataset into a training set consisting of 74 patients, utilizing threefold cross-validation for model development, and a test set of 17 patients to assess the model’s generalizability and robustness. We developed a deep-learning-based dynamic-aware model (DAM) to accurately predict responses to GC treatment. DAM uniquely addresses the challenge of interpreting complex patterns and temporal dynamics in treatment-response predictions by combining convolutional and fully connected neural networks for feature extraction with attention mechanisms for information integration. Specifically, it employs self-attention-based modules to integrate multi-object and multi-temporal data, as well as a cross-attention-based module to merge mismatched multisource dynamic data. Experimental results confirm the efficacy of DAM in deriving insights from longitudinal liquid biopsies and accurately predicting treatment responses. Furthermore, we identified six dynamic focus area features through DAM’s visual analysis and conducted preliminary studies to evaluate its potential for interpreting visual data from liquid biopsies.

## Methods

### Ethics

The Ethics Committee of Peking University Cancer Hospital granted ethical approval for this study (approval number: 2020KT08). All participants, or their legally authorized representatives, provided informed consent.

### Patients and data collection

**Fig. 1:**
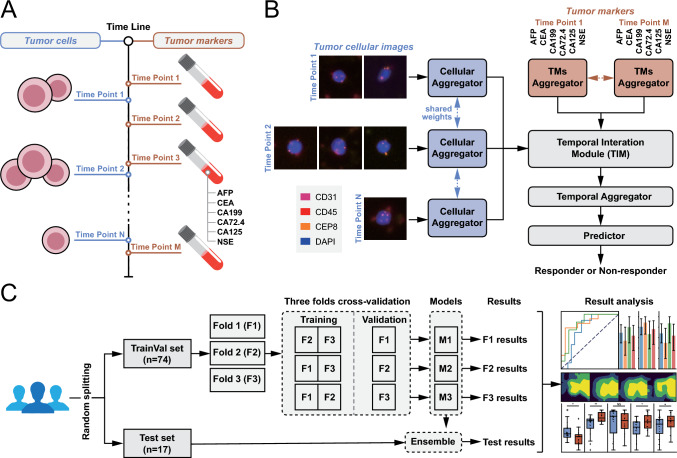
Workflow for predicting treatment response to GC using a dynamic-aware model (DAM) with longitudinal liquid biopsy data.** A** Data format: Tumor-related aneuploid cellular images and indices for six tumor markers (AFP, CEA, CA199, CA72.4, CA125, and NSE) were collected from longitudinal liquid biopsies at various time points, forming dynamic patient profiles. **B** Model framework: DAM comprises five components: The cellular aggregator and tumor marker aggregator compile tumor-related cellular images and tumor marker indices into distinct features, respectively. The temporal interaction module (TIM) integrates these mismatched multisource data. A temporal aggregator synthesizes this data into a patient-level feature, enabling the predictor to classify patients as responders or non-responders. **C** Experimental pipeline: The study included 91 patients with GC from Peking Cancer Hospital, encompassing 1895 tumor-related aneuploid cellular images and 1698 tumor marker indices obtained from longitudinal liquid biopsies. The patients were randomly divided into a training set of 74 patients and an independent test set of 17 patients. The training set was then subjected to threefold cross-validation (F1, F2, F3), resulting in three distinct trained models (M1, M2, M3) along with their respective validation results. Finally, these three trained models collaboratively predicted responses for the independent test set, and their predictions were ensembled to yield the final result

The study included patients diagnosed with GC from July 2019 to April 2022. For each patient, longitudinal liquid biopsies were collected, which comprised dynamic tumor-related aneuploid cellular images, including CTCs and CECs, as well as dynamic tumor marker indices, at baseline and during subsequent follow-ups (Figs. 1A and S1). Blood samples underwent density gradient centrifugation and microfluidic isolation to enrich for CTCs and CECs. Subsequently, the isolated cells were fixed onto slides and stained with specific markers, including cluster of differentiation 31 (CD31), cluster of differentiation 45 (CD45), centromere protein 8 (CEP8), and 4′,6-diamidino-2-phenylindole (DAPI), using an immunostaining-fluorescence in situ hybridization (iFISH) protocol according to the manufacturer’s protocol[54] (Cytelligen, San Diego, CA, USA) with minor alteration. The samples were then imaged using the automated Metafer-i•FISH® CTC 3D scanning and image analysis system [55], co-developed by Carl Zeiss (Oberkochen, Germany), MetaSystems (Altlussheim, Germany), and Cytelligen, to capture high-resolution multi-channel overlay images for analysis (more details are provided in Text S1). Furthermore, blood samples were analyzed in the laboratory to measure levels of various tumor markers, including alpha-fetoprotein (AFP), carcinoembryonic antigen (CEA), carbohydrate antigen 19-9 (CA19-9), cancer antigen 72-4 (CA72-4), cancer antigen 125 (CA125), and neuron-specific enolase (NSE), which constituted the collected tumor marker indices.

In patients with GC, treatment response is defined as follows: patients who achieve a complete response (CR) or a partial response (PR) according to the Response Evaluation Criteria in Solid Tumors (RECIST) are categorized as responders. Conversely, those exhibiting progressive disease (PD) or stable disease (SD) are classified as non-responders.

### The architecture of DAM

We introduced the DAM, a deep-learning-based dynamic-aware model designed to analyze dynamic tumor-related cellular images and tumor marker indices from longitudinal liquid biopsies to predict responses to GC treatment. Building on current research [56–61] that demonstrates the rich, multidimensional information contained in circulating tumor-related cellular images, this study employs advanced AI techniques to extract high-dimensional feature embeddings capable of capturing both inter-cell and inter-patient heterogeneity, going beyond traditional analyses that focus on single parameters such as copy number or cell counts. The architecture of the DAM, illustrated in Fig. 1B, consists of a comprehensive framework with five integrated components. The DAM begins with the cellular aggregator, which employs a ResNet-18 [38], as the feature extractor and a dual-stage Transformer [37], to create a unified representation of cellular data across various time points (Fig. S2). Concurrently, the tumor marker aggregator utilizes a dual-layer perceptron along with a similar dual-stage Transformer to integrate tumor marker indices over time (Fig. S3). Subsequently, the temporal interaction module (TIM) aligns these dynamic features using an advanced cross-attention mechanism (Fig. S4), ensuring effective synchronization and integration of mismatched multisource temporal data. Following this, the temporal aggregator employs a quad-stage Transformer to consolidate data from various time points into a comprehensive, patient-centric feature representation (Fig. S5). Finally, the predictor component utilizes a three-layer multilayer perceptron (MLP) to classify patients into responder or non-responder categories based on the integrated features (Fig. S6). All of these components work in concert to ensure a robust and accurate analysis of longitudinal liquid biopsies.

### Training and assessment procedures for DAM

This study meticulously designed experiments to ensure the effective training and fair verification of the DAM using a constrained dataset (Fig. 1C). The dataset was randomly divided, allocating 74 patients for training and reserving a separate set of 17 patients for independent testing. The training dataset was further subdivided into three subsets: fold-1 (F1), fold-2 (F2), and fold-3 (F3), comprising 26, 26, and 22 patients with GC, respectively. A threefold cross-validation strategy [62] was implemented, rotating these subsets between training and validation to fine-tune the model’s hyperparameters and architecture. This process yielded three distinct trained models, which collectively formed an ensemble model that was evaluated against the independent test set for robustness.

During model development, raw grid-arranged images were initially segmented into individual cellular images using a sliding window detection algorithm (see Fig. S11 for more details). Furthermore, during training, data augmentation techniques—including random horizontal and vertical flipping with a 50% probability, random rotations of up to 30°, as well as ImageNet RGB normalization—were applied to enhance model robustness and generalizability. In addition, all cell images were resized to 224 × 224 pixels to ensure consistent input dimensions for the convolutional neural network (CNN). To improve the model’s applicability in real-world medical scenarios, we introduced a dynamic longitudinal elimination strategy during training (Fig. S1). This strategy involved 80% non-repetitive random sampling along the temporal dimension of the dynamic tumor-related cellular images and dynamic tumor markers, effectively mimicking a data-level dropout. This strategy fosters model resilience and adaptability to practical challenges, such as missing temporal data. During validation and testing, we utilized all available longitudinal data without any random sampling to ensure a comprehensive assessment.

After training, we froze the model parameters for further analysis. We first applied the SoftMax activation function to the predictive probabilities of DAM and used these probabilities to plot receiver operating characteristic (ROC) curves and calculate the area under the curve (AUC) scores. Furthermore, by employing the Grad-CAM algorithm [63] on the final convolutional layer of the cellular aggregator, we produced insightful attention maps. Based on these maps, we quantified six dynamic features. Specifically, for a particular patient with a total of $N$ tumor-related cellular images $\{{I}_{1},{I}_{2},\dots ,{I}_{N}\}$ across $T$ time points, each image is associated with a corresponding attention map generated by Grad-CAM, denoted as $\{{A}_{1},{A}_{2},\dots ,{A}_{N}\}$. These attention maps were then subjected to binary segmentation using a 75-th percentile threshold to delineate focus areas $\{{F}_{1},{F}_{2},\dots ,{F}_{N}\}$, where the focus area is defined as the total area identified as foreground in the binary segmentation. The dynamic features, namely focus area variability (VarFA), minimum focus area (MinFA), maximum focus area (MaxFA), average focus area (AvgFA), and median focus area (MedFA), were denoted as follows:

$$\text{AvgFA}=\frac{1}{N}{\sum }_{i=1}^{N}{F}_{i},$$

$$\text{VarFA}=\frac{1}{N-1}{\sum }_{i=1}^{N}{\left({F}_{i}-AvgFA\right)}^{2},$$

$$\text{MinFA}=\text{min}\{{F}_{1},{F}_{2},\cdots ,{F}_{N}\},$$

$$\text{MaxFA}=\text{max}\{{F}_{1},{F}_{2},\cdots ,{F}_{N}\},$$

$$\text{MedFA}=\text{median}\left\{{F}_{1},{F}_{2},\cdots ,{F}_{N}\right\}.$$

In addition, focus area dispersion (DisFA) was quantified by determining the number of connected domains within the regions of interest, expressed as:

$$\text{DisFA}={\sum }_{i=1}^{N}{D}_{i},$$

where ${D}_{i}$ represents the number of connected domains in the $i$-th image. Finally, a three-layer MLP, was trained using these features to uncover nonlinear relationships between the features and treatment responses.

### Statistical analyses

**Fig. 2:**
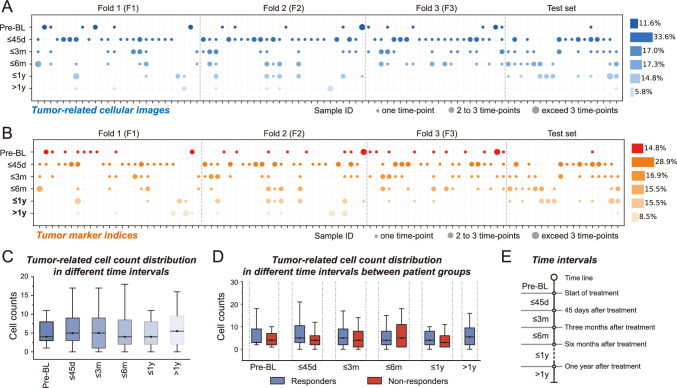
Visualization of longitudinal liquid biopsy data characteristics and dynamic distribution across patients with GC.** A** A bubble diagram shows the heterogeneity in tumor-related cellular images. Each column corresponds to an individual patient, while each row represents different time intervals: Pre-Baseline (Pre-BL), within 45 days post-baseline (≤45d), within 3 months post-baseline (≤3m), within 6 months post-baseline (≤6m), within a year post-baseline (≤1y), and beyond a year post-baseline (>1y). The presence of each dot signifies the number of time points collected from a patient within these respective time intervals, with larger dots indicating a higher frequency of sampling. An adjacent bar graph on the right further illustrates the sampling frequency across these time intervals. **B** A bubble diagram similarly depicts the heterogeneity in the distribution of tumor marker indices, employing an interpretation approach analogous to panel **A**. **C** A box plot highlights the heterogeneity in cell counts across the aforementioned time intervals. **D** A box plot illustrates the differences in cell count heterogeneity across time intervals between responders and non-responders. **E** A diagram illustrates the division of these time intervals

The sample size was contingent upon the number of patients fulfilling the inclusion criteria (first- or second-line treatment with data from at least two time points) rather than by a pre-established statistical methodology. The allocation of subjects across different groups, as delineated in Figs. 2C, D, 4A, C, was evaluated utilizing the Mann–Whitney U test. We performed our statistical analyses using R software (version 4.1.3) or Python (version 3.7.10). A *P* value threshold of less than 0.05 was established as the criterion for statistical significance.

To ensure the reproducibility of this study, comprehensive methodologies and data management protocols are thoroughly documented in the supplementary information (see Figs. S1–S11 and Texts S1–S3). The robustness of DAM was augmented through a threefold cross-validation strategy and further validated using an independent test set. Moreover, the source code, implemented by PyTorch [64], is available in the supplementary materials.

### Role of funders

The funders played no role in the study design, data collection, data analysis, data interpretation, or the writing of the report.

## Results

### Patient profile and data characteristics

The data for this study were collected from patients with GC treated at Peking Cancer Hospital between July 2019 and April 2022. The dataset comprises longitudinal liquid biopsy data from 91 patients, encompassing 1895 aneuploid tumor-related cellular images and 1698 tumor marker indices. Among these patients, 8 received chemotherapy, 5 received targeted therapy, and 78 received immunotherapy. In addition, 72 patients were treated in the first-line setting, while 19 were treated in the second-line setting. For all patients, the first liquid biopsy was collected during the early phase of treatment—typically within the first 3 months. Moreover, for most patients, subsequent dynamic samples were collected at intervals of less than 3 months. The dataset was randomly divided into a training set of 74 patients for deep-learning model development, utilizing threefold cross-validation, and a test set of the remaining 17 patients to assess the model’s generalizability and robustness (Table 1). The median age of the patients was 65 years, with an interquartile range of 57 to 72 years. Males constituted 80.22% of the dataset, indicating a pronounced male predominance. Treatment lines included first-line therapies (80.22%) and other-line therapies (19.78%). Most patients were diagnosed with advanced stage disease.

**Table 1:** Pathologi characteristics of enrolled patients

Patient baseline characteristics		*N* (%)					*P*
		Fold-1	Fold-2	Fold-3	Test		
Age							
≥60		12 (46.2)	17 (65.4)	12 (54.5)	11 (64.7)		0.482
<60		14 (53.8)	9 (34.6)	10 (45.5)	6 (35.3)		
Sex							
Male		22 (84.6)	20 (76.9)	20 (90.9)	11 (64.7)		0.199
Female		4 (15.4)	6 (23.1)	2 (9.1)	6 (35.3)		
Location							
GEJ		9 (34.6)	8 (30.8)	7 (31.8)	5 (29.4)		0.985
Non-GEJ		17 (65.4)	18 (69.2)	15 (68.2)	12 (70.6)		
Differentiation							
High		0 (0.0)	1 (3.8)	1 (4.5)	0 (0.0)		0.562
Moderate-high		0 (0.0)	0	1 (4.5)	0 (0.0)		
Moderate		11 (42.3)	13 (50.0)	6 (27.3)	6 (35.3)		
Moderate-poor		7 (26.9)	5 (19.2)	6 (27.3)	2 (11.8)		
Poor		8 (30.8)	7 (26.9)	7 (31.8)	9 (52.9)		
Unknown		0 (0.0)	0 (0.0)	1 (4.5)	0 (0.0)		
Lauren classification							
Intestinal type		17 (65.4)	17 (65.4)	14 (63.6)	13 (76.5)		0.818
Diffused type		4 (15.4)	2 (7.7)	3 (13.6)	3 (17.6)		
Mixed type		4 (15.4)	4 (15.4)	4 (18.2)	1 (5.9)		
Unknown		1 (3.8)	3 (11.5)	1 (4.5)	0 (0.0)		
HER2 expression (IHC)							
Positive		17 (65.4)	18 (69.2)	17 (77.3)	13 (76.5)		0.775
Negative		9 (34.6)	8 (30.8)	5 (22.7)	4 (23.5)		
Therapeutic efficacy							
CR		0 (0.0)	1 (3.8)	0 (0.0)	0 (0.0)		0.568
PR		17 (65.4)	13 (50.0)	15 (68.2)	8 (47.1)		
SD		4 (15.4)	7 (26.9)	6 (27.3)	7 (41.2)		
PD		5 (19.2)	5 (19.2)	1 (4.5)	2 (11.8)		

*GEJ* esophagogastric junction, *IHC* immunohistochemical, *CR* complete response, PR partial response, *SD* stable disease, *PD* progressive disease

The study meticulously tracked the temporal distribution of liquid biopsy metrics to capture the dynamic aspects of GC progression. Figure 2A and B show the dynamic distribution of tumor-related cellular images and tumor marker indices across patients and time intervals, respectively. The median number of collection time points per patient was three for both tumor-related cellular images and tumor marker indices, with an interquartile range of 1 to 4. This median indicates robust longitudinal data collection, providing a solid foundation for analyzing disease progression. In addition, Fig. 2C highlights fluctuations in tumor-related cell counts over different time intervals (Fig. 2E), revealing consistent distribution patterns across all intervals. This consistency, observed regardless of treatment-response (Fig. 2D), underscores the nuanced complexity of disease trajectories as reflected in liquid biopsy profiles.

### Enhanced response prediction using the dynamic-aware model compared to the cell-counting-based model

**Fig. 3:**
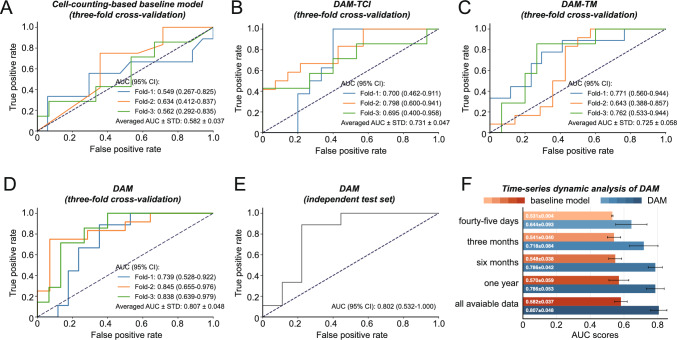
Comparative performance analysis of models predicting response to GC treatment. **A** The receiver operating characteristic (ROC) curve for the cell-counting-based baseline model, which uses cell counts to predict treatment responses (threefold cross-validation). **B, C** ROC curves for two DAM-variants: DAM-TCI and DAM-TM. **D** The ROC curve for the proposed DAM. **E** Robustness of the proposed DAM on an independent test set. **F** A time-series dynamic analysis of the baseline model and DAM was performed for periods ranging from 45 days to the full available data. The black lines on the bar chart represent the standard deviation

A comprehensive analysis shows that the proposed DAM outperforms the traditional cell-counting-based model in predicting response of GC treatment (Fig. 3). The ROCs from threefold cross-validation shows that fold-1 achieved an AUC of 0.739 (95% Confidence Interval [CI]: 0.528–0.922), fold-2 achieved an AUC of 0.845 (95% CI: 0.655–0.976), and fold-3 achieved an AUC of 0.838 (95% CI: 0.639–0.979) (Fig. 3D). The overall average AUC inclusive of the standard deviation, was 0.807 ± 0.048. Furthermore, when applied to an independent test set, the DAM demonstrated robust performance with an AUC of 0.802 (95% CI: 0.532–1.000; Fig. 3E). In comparison, the cell-counting model, which utilized the XGBoost algorithm based on baseline tumor-related cell counts, recorded an average AUC of 0.582 ± 0.037 (Fig. 3A and Table S1).

To examine the impact of two longitudinal data sources—dynamic tumor-related cellular images and dynamic tumor markers—we developed two DAM variants: DAM-TCI and DAM-TM. DAM-TCI focuses exclusively on utilizing dynamic tumor-related cellular images to predict treatment response, while DAM-TM concentrates solely on dynamic tumor markers. In our experiments, DAM-TCI achieved an average AUC of 0.731 ± 0.047 (Fig. 3B), whereas DAM-TM achieved an average AUC of 0.725 ± 0.058 (Fig. 3C). Both variants outperformed the cell-counting-based model, highlighting the effectiveness of DAM’s dynamic design in synthesizing data from different time points.

The discrepancies in collection times for dynamic tumor-related cellular images and tumor marker indices (Fig. S1) necessitate the model’s ability to adaptively integrate mismatched multisource dynamic data. We proposed an enhanced cross-attention module (TIM) to address this challenge. By integrating dynamic tumor-related cellular images with dynamic tumor marker indices, TIM improved performance to 0.807 (Fig. 3D), surpassing the individual dynamic data sources. This outcome underscores the efficacy of TIM in amalgamating multiple dynamic data types and highlights the complementary role of tumor marker information in enhancing tumor-related cellular images.

Moreover, ablation experiments indicated that replacing or removing the proposed modules with simpler operations led to a decrease in performance. As shown in Fig. S12, a contrast model utilizing a classic LSTM-based approach for dynamic modeling [52, 53] achieved an average AUC of 0.635, while a straightforward multimodal fusion method employing vector concatenation and summation reached average AUCs of 0.760 and 0.777, respectively. In contrast, our method, which incorporates the cell and temporal aggregator with the TIM module, attained an average AUC of 0.807. These results validate the critical role of the proposed modules in optimally integrating longitudinal liquid biopsy data. From a training perspective, to address the challenge of limited data samples, we introduced two data-related strategies—data augmentation and dynamic longitudinal elimination—during model development. As illustrated in Fig. S13, employing either of these strategies independently improved the AUC (from 0.755 to 0.773 or 0.770), but applying both strategies simultaneously yielded the highest performance, achieving an average AUC of 0.807. The removal of either strategy resulted in a decrease in the model’s robustness and AUC, underscoring the necessity of both approaches working in tandem to achieve optimal predictive performance. In addition, as illustrated in Fig. S14, an ablation experiments demonstrate that the current collection of cellular images effectively captures inter-patient heterogeneity, confirming that our dataset adequately reflects the biologic diversity of tumors.

Subsequently, we performed a time-series dynamic analysis of DAM, allowing the model to access data from specified time intervals, including 45 days (45d), 3 months, 6 months, 1 year, and all available data (all) in the collection (Fig. 2E). The performance of DAM gradually improved with extended time intervals, demonstrating average AUCs of 0.644 (45 days), 0.718 (3 months), 0.786 (6 months), 0.786 (1 year), and 0.807 (all). This indicates that including more dynamic information from extended treatment records enhances prediction accuracy. Notably, thanks to the flexible dynamic temporal encoding in the TIM, the temporal aggregator, and the dynamic longitudinal elimination strategy employed during the training phase, DAM can function as an early predictor at inference time when only early-treatment dynamic data are provided. As illustrated in Fig. 3F, when we restrict the model to early-treatment dynamic data collected within 3 months post-baseline, DAM achieves a response prediction AUC of 0.718, compared to an AUC of only 0.541 for the cell-count-based baseline model. This highlights DAM’s capability to serve as an early-treatment-response predictor. Furthermore, DAM exhibited a substantial performance improvement within the first 6 months of treatment, with an absolute AUC increase of 0.142 (from 0.644 to 0.786). A notable portion of this gain was observed within the initial 3-month early-treatment window, with an absolute AUC increase of 0.074 (from 0.64 to 0.718). The performance improvement beyond 6 months was comparatively smaller, with an absolute AUC increase of 0.021 (from 0.786 to 0.807). These results collectively underscore DAM’s effectiveness in utilizing early treatment data for accurate treatment-response prediction, aligning with the critical clinical need for the prompt identification of non-responders during the treatment process.

### Visual analysis of DAM

**Fig. 4:**
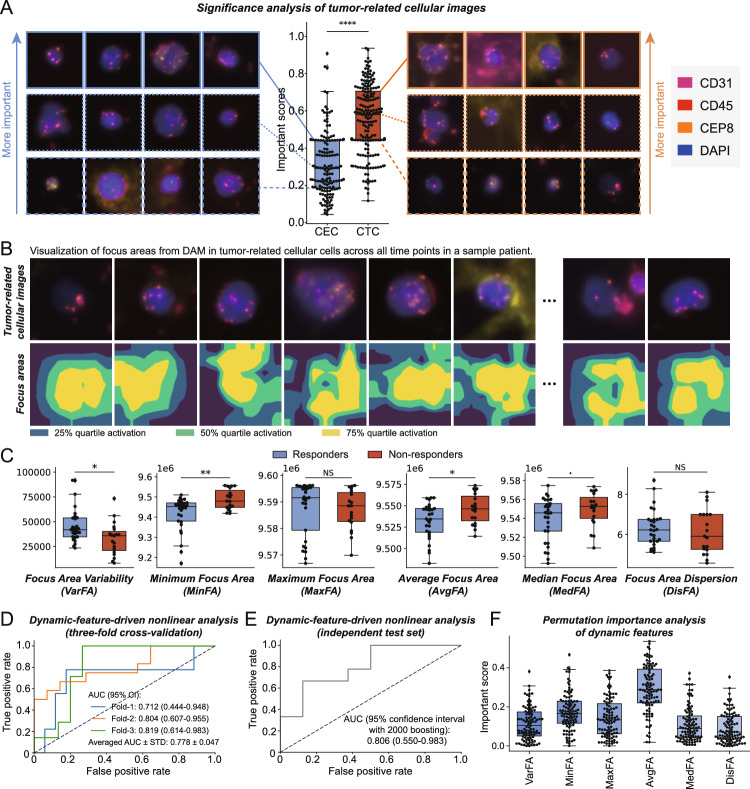
Visual analysis of DAM’s attention mechanisms and focus areas.** A** A box plot showcases the distribution of importance scores assigned to cellular features within the cellular aggregator, grouped by CTCs and CECs. **B** Visualization of focus areas identified by the model in tumor-related cellular images across various time points, showing different quartile activations. **C** Box plots illustrating the statistical distribution of six dynamic focus-area-related features for responders and non-responders. **D** ROC curves of a dynamic-feature-driven nonlinear analysis using an MLP (threefold cross-validation). **E** Robustness evaluation of the nonlinear analysis on the independent test set. **F** Permutation importance analysis for six dynamic focus-area-related features

The previously employed cell-counting approach neglects the visual characteristics of tumor-related cellular images. In contrast, the proposed DAM enhances the interpretation of complex visual data, enabling a more comprehensive analysis of images. We explored DAM’s visual focus mechanism by analyzing the distributions of important scores for cellular features within the aggregator, categorized by CTCs and CECs (Fig. 4A). CTCs exhibited significantly higher important scores than CECs (two-sided Mann–Whitney U test, *P* < 0.0001), emphasizing the crucial role of CTCs in DAM. Consequently, we visualized tumor-related cellular images at the 25-th, 50-th, and 75-th percentiles of the important score distribution. Our intuitive observations suggest that larger and more semantically complex CTCs have a more substantial impact on outcomes. This leads us to conclude that DAM primarily interprets visual information from liquid biopsies through two key visual signals: size and heterogeneity within the image.

Furthermore, by utilizing the Grad-CAM algorithm, we visualized DAM’s visual encoder, noting variations in the sizes and dispersion of focus areas across different cellular images (Fig. 4B). Building on the insights gained from DAM’s visual focus analysis, we further explored dynamic focus-area-related visual features (for more details, see the Methods section), including VarFA, MinFA, MaxFA, AvgFA, MedFA, and DisFA (Fig. 4C). Moreover, Fig. S15 displays the distribution of these dynamic features across patients, highlighting considerable inter-patient heterogeneity. This observation underscores the effectiveness of our AI-driven model in capturing patient-specific differences from longitudinal liquid biopsy data, thereby enhancing its potential to predict treatment responses by accounting for this variability. Consequently, we further employed a nonlinear analysis using a three-layer MLP to predict treatment response based on these dynamic visual features (Fig. S6), yielding AUC scores of 0.712 (95% CI: 0.444–0.948) in fold-1, 0.804 (95% CI: 0.607–0.955) in fold-2, and 0.819 (95% CI: 0.614–0.983) in fold-3, with an average score of 0.778 ± 0.047 (Fig. 4D). This performance is comparable to that of DAM on the test set (0.806 vs. 0.802; Fig. 4E). Moreover, a permutation importance analysis highlighted AvgFA as particularly important, underscoring tumor cell size’s importance in DAM. These analyses offer initial insights into the visual characteristics of tumor-related cellular images, suggesting that deep-learning models like DAM have the potential to summarize generalized spatial visual information.

## Discussion

GC remains a significant global health challenge, underscoring the need for precise and early evaluation of treatment effectiveness. Recent studies on liquid biopsy data, including CTCS, CECs, and tumor markers, have highlighted their potential in cancer treatment management. Analyzing liquid biopsy data offers two main advantages: it provides crucial cellular-level biomarkers and is more convenient than traditional methods such as CT scans and histopathology. This convenience facilitates the acquisition of time-sensitive data. However, a notable research gap exists in processing and interpreting complex, longitudinal liquid biopsy data, highlighting the need for more advanced analytical methods. Our study is the first to apply longitudinal liquid biopsy data in the development of an innovative AI model, DAM. DAM utilizes cutting-edge AI technology to integrate multisource, multi-object, and multi-temporal data, enabling accurate response predictions and signifying a considerable advancement in the field.

A key finding that distinguishes our research from previous liquid biopsy studies is DAM’s superior performance in modeling longitudinal liquid biopsies for predicting treatment responses. Unlike earlier methods that relied on cell counting, DAM processes dynamic data from CTCs, CECs, tumor markers, and associated visual imagery concurrently. The comparative analysis underscores DAM’s enhanced capability in predicting responses to GC treatment, and an evaluation using an independent test set reveals its robustness. In addition, two variants of DAM, DAM-TCI and DAM-TM, underscore the importance of longitudinal information and illustrate the effectiveness of dynamic modeling within DAM. The analysis of the impact of dynamic data from diverse treatment time intervals on efficacy prediction reveals that DAM can accurately forecast outcomes using early treatment data, aligning with clinical usage scenarios.

This study also showcases the exceptional flexibility and expansiveness of the DAM module design, which adeptly aggregates diverse elements, such as tumor-related cellular images. In addition, the TIM within DAM demonstrates exceptional proficiency in aligning temporally mismatched data from diverse dynamic sources. By utilizing tumor markers to enhance the analysis of tumor-related cellular images, TIM establishes a groundbreaking approach to data integration. This method is particularly well-suited for medical contexts, where temporal discrepancies between datasets are common. Its applicability to future scenarios suggests broad utility beyond the immediate context, offering a promising avenue for integrating diverse data types across a wide range of fields. Besides, within DAM, the temporal aggregator skillfully manages a varied temporal distribution of dynamic data. This innovative design not only accommodates multiple objects, times, and modalities but also seamlessly incorporates non-dynamic (single-time-point) data, such as historical radiologic and pathologic records. The versatility and scalability inherent in the module design are crucial, facilitating the adaptation and integration of a wide spectrum of data types and sources, thereby enhancing the model’s applicability and robustness in complex clinical scenarios.

By leveraging advanced deep-learning technologies such as CNNs and Transformers, DAM effectively models visual information from tumor-related cellular images, a dimension often overlooked by traditional cell-counting methods. This approach facilitated a preliminary analysis of DAM’s visual attention patterns. Through detailed evaluations of areas of interest identified by DAM within the images, we discerned six dynamic features that are significantly relevance to treatment response. These insights highlight the model’s profound capacity to understand and interpret visual information while also showcasing DAM’s intricate structural design. In this context, the visual encoder skillfully extracts key image features, while the transformer-based feature aggregation efficiently manages the diverse distributions present within and among cellular images. This advanced methodology holds substantial promise for future explorations into deeper visual understanding patterns in AI models, providing invaluable assistance to physicians in interpreting complex visual information, thereby enhancing diagnostic and therapeutic approaches in oncology.

However, two notable limitations of DAM are as follows: (1) Although we have collected all available longitudinal data to the best of our ability and validated the feasibility of using advanced AI models to predict GC treatment responses, the current dataset encompasses a variety of treatment regimens for patients with GC. As more data accumulates, detailed evaluations of treatment-specific efficacy and further multi-center validation will be essential to explore our model’s applicability within specific treatment contexts and to thoroughly investigate its performance as an early-response predictor. (2) While the current module design effectively manages dynamic liquid biopsy data and achieves multimodal integration, future data collection should not only focus on expanding the overall sample size but also on enriching the horizontal data for each patient. For instance, increasing the number of tumor-related cellular images per patient and incorporating additional dynamic data modalities—such as temporal CT scans and clinical report texts—will be crucial for fully exploring the model’s potential in capturing a more comprehensive spectrum of both inter- and intra-tumor heterogeneity.

## Conclusions

In summary, this study represents a pioneering effort to utilize AI technology to dynamically model longitudinal liquid biopsy data, marking a notable advancement toward precise response prediction in patients with GC. This research demonstrates the potential of AI in analyzing longitudinal liquid biopsies for precision medicine and highlights its impressive ability to comprehend complex visual features, promising enhanced collaboration between AI and clinicians in clinical settings.

## Supplementary Information

Below is the link to the electronic supplementary material.

Supplementary file1 (DOCX 7207 KB)

## Data Availability

The datasets will be available upon reasonable request via email to the corresponding author (zhangli_pku@pku.edu.cn). The code for the model development can be found in the Supplementary Materials.

## Abbreviations

GC: Gastric cancer
AI: Artificial intelligence
CTC: Circulating tumor cell
CEC: Circulating endothelial cell
CT: Computerized tomography
TIM: Temporal interaction module
MLP: Multilayer perceptron
CNN: Convolutional neural network
DAM: Dynamic-aware model
ROC: Receiver operating characteristic
AUC: Area under the curve
AFP: Alpha-fetoprotein
CEA: Carcinoembryonic antigen
CA19-9: Carbohydrate antigen 19-9
CA72-4: Cancer antigen 72-4
CA125: Cancer antigen 125
NSE: Neuron-specific enolase
CR: Complete response
PR: Partial response
PD: Progressive disease
SD: Stable disease
RECIST: Response Evaluation Criteria in Solid Tumors
