# Supplementary material for: Predicting response to patients with gastric cancer via a dynamic-aware model with longitudinal liquid biopsy data
Source: Gastric Cancer. 2025 Jun 17;28(5):886–98. doi: 10.1007/s10120-025-01628-4 (PMC12378481; doi:10.1007/s10120-025-01628-4)
Supplement: Supplementary file 1 — Supplementary file1 (DOCX 7207 KB) [file 10120_2025_1628_MOESM1_ESM.docx]

*Supplementary information*

**Predicting response to gastric cancer patients via a dynamic-aware model with longitudinal liquid biopsy data**

Zifan Chen et al.

Supplementary Figures

## Fig. S1. Input samples.

Visualization of tumor cells and tumor markers in three individual patients: Patient A with Stable Disease (SD), Patient B with Progressive Disease (PD), and Patient C with Partial Response (PR).

## Fig. S2. The details of the cellular aggregator.

The cellular aggregator module takes its input from a series of cellular images at a specific time point $T$. Assuming there are $N$ cellular images at this time point. For the $n^{th}$ image, it is represented as $I_{n}\in\mathbb{R}^{M\times N}$. A feature extractor $\mathcal{F}$, specifically ResNet-18^1^, is employed to map each image $I_{n}$ into a corresponding feature embedding ${\mathcal{F}\left( I_{n} \right)=F}_{n}\in\mathbb{R}^{C}$, resulting in $N$ such embeddings. To facilitate information aggregation, we introduce an extended token (depicted in blue), akin to the classification token used in Vision Transformer (ViT)^2^. This forms a sequence input $\in\mathbb{R}^{\left( N+1 \right)\times C}$ for the Multi-Head Attention (MHA) mechanism^3^. The workings of MHA are detailed in the right sub-figure, the input sequence is initially divided into $H$ heads to perform parallelized self-attention computations. Subsequently, these features are mapped into three functional features—Key ($K$), Query ($Q$), and Value ($V$)—via three fully connected (FC) layers. With each head, the Query and Key are matrix-multiplied to generate an attention score map ($QK^{T}$), which is then scaled and normalized using the SoftMax function. The Value feature is multiplied by this normalized attention score map to yield the output for each head. The outputs from all heads are concatenated to form the final result $\in\mathbb{R}^{\left( N+1 \right)\times C}$. We repeated the MHA two times in our model. Thanks to the self-attention operation, the extended token effectively captures information from all other tokens (cellular images), allowing it to serve directly as the aggregated feature.

## Fig. S3. The details of the tumor marker aggregator.

The tumor marker (TM) aggregator module takes its input from a series of tumor marker indices at a specific time point $T'$. Assuming there are $M$ tumor markers at this time point, typically including AFP, CEA, CA199, CA72.4, CA125, and NSE, each marker is represented by a numerical value. Initially, these values are normalized by their corresponding pre-computed mean and variance, as detailed in Table SXX. Subsequently, they are fed into a TM feature extractor, which comprises two Fully Connected (FC) layers, each followed by ReLU activation functions. Notably, during the training phase, the second FC layer undergoes a dropout of 25% of its nodes to prevent overfitting. Similar to the cellular aggregator, an extended token (depicted in blue) is introduced and combined with the extracted TM features to form the input sequence for the Multi-Head Attention (MHA) mechanism. This MHA process is iteratively performed four times to comprehensively capture the interrelationships among different tumor markers, ultimately yielding the aggregated feature.


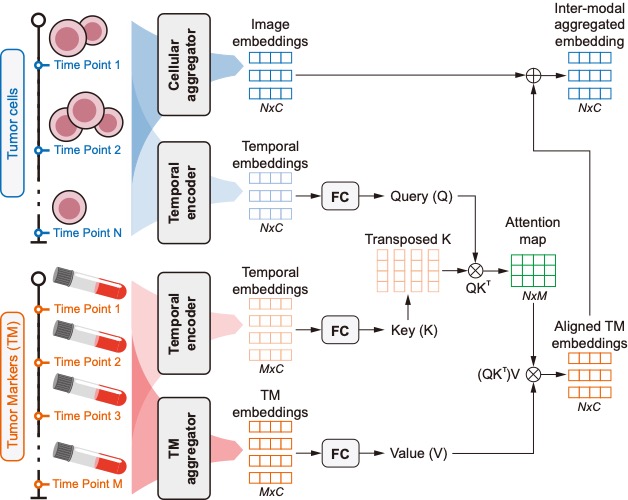


## Fig. S4. The details of the temporal interaction module (TIM).

TIM is engineered to align the information from tumor cells and tumor markers across disparate time points. Assuming that tumor cells are sampled at $N$ time points (for this example, $N=3$). Using the cellular aggregator (Figure S2), we obtain image embeddings $\in\mathbb{R}^{N\times C}$. Concurrently, we employ a temporal encoder, inspired by the cosine coding for position embedding as introduced in the previous study^3^, to map temporal information—specifically, the months elapsed since baseline treatment—into a series of temporal embeddings $\in\mathbb{R}^{N\times C}.$ For tumor markers, the TM aggregator is used to derive their TM embeddings $\in\mathbb{R}^{M\times C}$ from M time points. A similar temporal encoder is employed to generate their corresponding temporal embeddings $\in\mathbb{R}^{M\times C}$. Subsequently, the temporal embeddings for the tumor markers act as the Key, while the image temporal embeddings serve as the Query. Matrix multiplication between these two sets of embeddings yields an attention map $\in\mathbb{R}^{N\times M}$. The TM embeddings are treated as the Value and are multiplied by this attention map, resulting in aligned TM embeddings $\in\mathbb{R}^{N\times C}$. Finally, we compute the average of the image embeddings and the aligned TM embeddings to obtain the inter-modal aggregated embedding $\in\mathbb{R}^{N\times C}$.

## Fig. S5. The details of the temporal aggregator and the predictor.

The output from the temporal interaction module (TIM) is an inter-modal aggregated embedding $\in\mathbb{R}^{N\times C}$. This output is then processed by the temporal aggregator to consolidate the temporal information. Similar to the cellular aggregator and the tumor marker aggregator, an extended token (depicted in blue) is introduced to facilitate the aggregation of temporal information through the multi-head attention (MHA) mechanism. The MHA process is iteratively executed four times to fully capture the temporal dynamics, resulting in a patient-level aggregated feature. This aggregated feature serves as the input to the predictor module, which comprises two fully connected layers and is tasked with generating the final treatment response prediction.

## Fig. S6. The DynFAFeat-driven Multilayer Perceptron (MLP).

The DynFAFeat-driven MLP features a sequence of layers beginning with an input linear layer that expands a six-dimensional DynFAFeat into a 16-dimensional feature space. This is immediately followed by a ReLU activation function to introduce non-linearity. To mitigate the risk of overfitting, a dropout layer with a 20% dropout rate is applied. The network continues with another 16-dimensional linear layer coupled with a ReLU activation function for further non-linear transformation. A second dropout layer, also with a 20% dropout rate, is employed to enhance the model's generalization capabilities. The architecture concludes with a final linear layer that compresses the feature space down to two dimensions, which could be used for predicting responder or non-responder. The training and evaluation of the DynFAFeat-driven MLP is the same as DAM with a three-fold cross-validation for developing the model and an independent test set for robustness assessment.


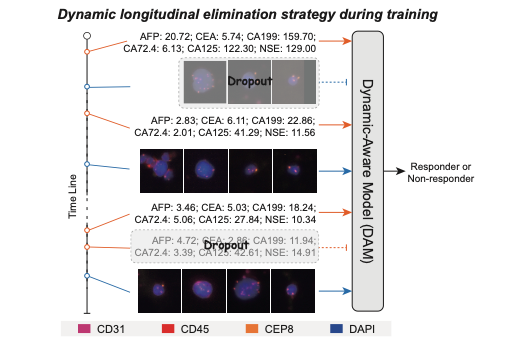


## Fig. S7. The dynamic longitudinal elimination strategy.

The timeline contains longitudinal liquid biopsies, with each time point showcasing corresponding tumor markers or cellular images. The strategy incorporates an 80% non-repetitive random sampling along the temporal dimension of the tumor cells and tumor markers, which serves to emulate a data-level dropout (as shown in the dashed line transparent rectangle). This methodology enhances the model's resilience and adaptability to potential temporal inconsistencies and instances of missing data. During training, DAM utilizes the selectively sampled data for prediction. For validation and testing phases, DAM employs all available longitudinal data without any random sampling to allow for a comprehensive assessment.

**
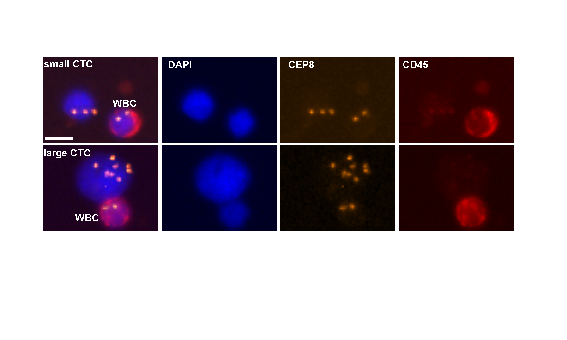
**

## Fig. S8. Visualization of CTC size heterogeneity and associated markers.

Displayed here are representative images of both small and large circulating tumor cells (CTCs) alongside a white blood cell (WBC) for size comparison. The leftmost column showcases composite images with distinct staining for both the CTC and WBC. Subsequent columns present staining patterns for DAPI (indicative of nuclei), CEP8 (a marker indicative of chromosome 8), and CD45 (a pan-leukocyte marker). The differential size and marker expression between small and large CTCs emphasize the inherent variability within CTC populations, highlighting the importance of comprehensive characterization in oncological studies.


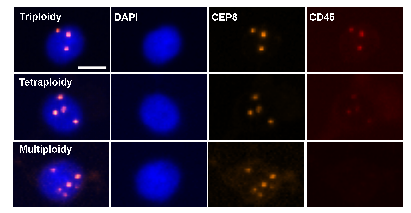


## Fig. S9. Characterization of Tumor Marker Ploidy Variations.

Presented are representative images illustrating variations in ploidy: triploidy, tetraploidy, and multiploidy. The leftmost column highlights the overall staining patterns for each ploidy level. The subsequent columns detail specific staining for DAPI (representing nuclei), CEP8 (a marker indicative of chromosome 8), and CD46 (an immune-related marker). The distinctive patterns and intensities observed for each ploidy type underscore the genetic heterogeneity and complexity of tumor cell populations, emphasizing the value of precise ploidy determination in oncological assessments.

**
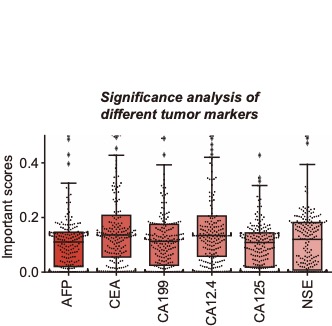
**

## Fig. S10. Significance evaluation across six tumor markers.

This section displays the important scores related to various tumor markers: AFP, CEA, CA199, CA72-4, CA125, and NSE. Boxplots represent the range, median, and interquartile ranges of the scores. The superimposed black swarmplots show individual data points, with outlier observations highlighted. The scale range of y is limited between 0.0 and 0.5. While all markers are pertinent, there are differences in their significance. Specifically, CEA and CA72.4 have higher median importance scores, emphasizing their key roles in DAM. On the other hand, markers such as CA199 and NSE show varied scores, indicating their inconsistency across gastric cancer patients. In essence, while each tumor marker is essential, CEA and CA72.4 are particularly crucial for predicting within DAM.


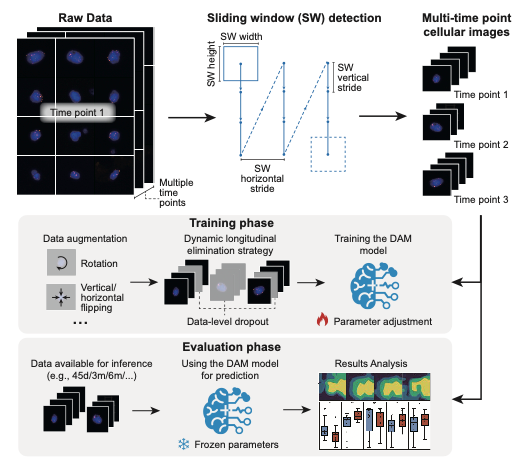


## Fig. S11. Image data preprocess workflow.

The workflow begins with raw cellular images collected at multiple time points. A sliding window (SW) detection strategy is used to segment cellular image patches. During training phase, data augmentation (e.g., rotation and flipping) is then applied, followed by a dynamic longitudinal elimination strategy to enhance the model’s effectiveness and robustness. In the evaluation phase, the trained DAM is frozen and applied to available multi-time point data for prediction. The resulting outputs are analyzed to assess model performance and draw biological insights.


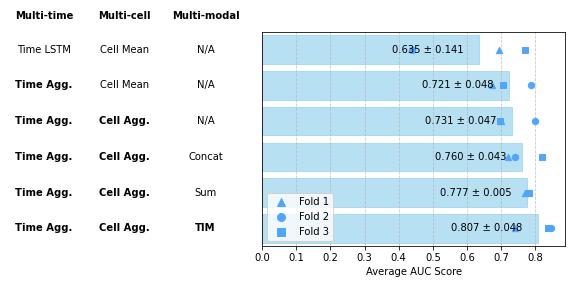


## Fig. S12. Ablation study on the impact of proposed modules in model construction and performance enhancement.

This figure presents the results of an ablation study designed to evaluate how each proposed module (highlighted in bold) contributes to overall model performance. Different model configurations are compared across three dimensions—multi-time, multi-cell, and multi-modal—using average AUC scores with standard deviations.


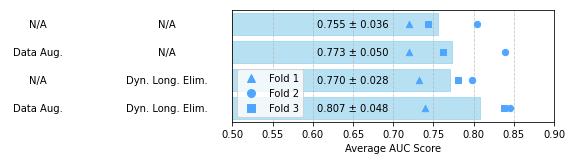


## Fig. S13. Ablation study of training strategies with data augmentation and dynamic longitudinal elimination.

This figure presents an ablation study examining the effects of two applied training strategies—data augmentation (Data Aug.) and dynamic longitudinal elimination (Dyn. Long. Elim.)—on model performance. Four configurations are compared, reflecting the presence or absence of each strategy. The average AUC scores with standard deviations are shown across three folds.

## Fig. S14. Effect of cellular image sampling percentage on DAM model performance.

This figure illustrates how varying the number of cellular images per patient influences the model’s performance. For each time point and patient, we sampled cellular images at different percentages to simulate a range of average image counts (ensuring that at least one image was used if the computed sample was less than one) and repeated the experiment five times. The colored dots represent individual AUC scores from these repetitions, while the dashed line and numeric labels indicate the mean AUC at each sampling percentage. Error bars denote the 25^th^–75^th^ percentile range, highlighting the variability in model performance across repeated trials.

## Fig. S15. Distribution of dynamic focus-area-related features across patients.

This figure illustrates the inter-patient heterogeneity in the distribution of dynamic features. Each shape represents a distinct dynamic feature, while colors indicate non-responders (red) and responders (blue) to GC treatment.

Supplementary Tables

## Table S1. Comparative performance of count-based machine learning models.

The table provides a detailed overview of the performance of various machine learning models, including SVM, Tree, LogReg, RF, and XGBoost, using cell count as the primary feature. Within the validation section, the first three columns represent the AUC scores for the respective folds (F1, F2, F3), while the fourth column illustrates the average AUC, accompanied by its standard deviation.

| Model | Three-fold cross-validation | | | |
| --- | --- | --- | --- | --- |
|  | F1 | F2 | F3 |  |
| SVM | 0.526 | 0.506 | 0.538 | 0.523$\pm$0.013 |
| Tree | 0.634 | 0.530 | 0.557 | 0.574$\pm$0.044 |
| LogReg | 0.408 | 0.500 | 0.467 | 0.458$\pm$0.038 |
| RF | 0.549 | 0.634 | 0.562 | 0.582$\pm$0.037 |
| XGBoost | 0.503 | 0.628 | 0.524 | 0.552$\pm$0.55 |

- SVM=Support Vector Machine, implemented by sklearn.svm.SVC(C=0.6, kernel=’rbf’, gamma=20, decision_function_shape=’ovr’)
- Tree=Decision-making Tree, implemented by sklearn.tree.DecisionTreeClassifier()
- LogReg=Logistic Regression, implemented by sklearn.linear_model.LogisticRegression()
- RF=Random Forest, implemented by sklearn.ensemble.RandomForestRegressor()
- XGBoost, implemented by xgboost.XGBRegressor()
- F1=Using Fold-1 as the validation set, and Fold-2 and Fold-3 as the training set.
- F2=Using Fold-2 as the validation set, and Fold-1 and Fold-3 as the training set.
- F3=Using Fold-3 as the validation set, and Fold-1 and Fold-2 as the training set.

Supplementary Texts

## Text S1. Tumor related cells detection by SE-iFISH

Briefly, 6 ml of peripheral blood was collected into a tube containing ACD anti-coagulant (Becton Dickinson, Franklin Lakes, NJ, USA). Blood samples were centrifuged at 400 g for 15 min at room temperature. The upper layer of plasma was discarded and 1×Concentrated CRC buffer was gently added to 6ml, then invert and mix well. The above mixture containing sedimented blood cells loaded onto 3ml of the non-hematopoietic cell separation matrix in a 50 ml tube, and subsequently centrifuged at 350 g for 6 min. The entire solution containing WBCs and tumor cells above the red blood cell (RBC) layer was collected into a 50 ml tube, and subsequently incubated with 300 μl of immuno-magnetic beads buffer conjugated to a cocktail of anti-leukocyte mAbs at room temperature for 20 min in the horizontal shake bed. WBCs bound to immuno-beads were depleted using a magnetic separator. Solutions free of magnetic beads were collected, washing and spun twice at 500 g for 5 min and 400g for 5min. 100 μl of sedimented cells subjected to subsequent iFISH.

The subtraction enriched cells were subjected to liquid immunofluorescence staining. Samples were subsequently incubated with anti-CD45 monoclonal antibody conjugated to Alexa Fluor (AF) 594 and anti-CD31 monoclonal antibody conjugated to Alexa Fluor (AF) 488. After washing and spun, the suspended cells were mixed with cytelligen fixative to fix cells and applied to the specimen frame of Cytelligen CTC slides.

Dried monolayer cells on the coated CTC slides were hybridized with centromere probe 8 Spectrum Orange (CEP8, Vysis and Abbott Laboratories, Chicago, IL, USA). Lastly, samples were mounted with mounting media and subjected to the automated Metafer-iFISH® CTC 3D scanning to capture high-resolution multi-channel overlay images for analysis. The image analysis system co-developed by Carl Zeiss (Oberkochen, Germany), MetaSystems (Altlus- sheim, Germany) and Cytelligen. CTCs were defined as DAPI+, CD45- and CD31- with aneuploid Chr8. The characteristic of CTECs were DAPI+, CD45- and CD31+ with aneuploid Chr8.

## Text S2. Implementation details.

**Running environment configuration**: We implemented our model using PyTorch^4^ (version 1.11.0) powered by CUDA 11.3. All experiments were run in one NVIDIA GeForce RTX 2080 Ti GPU with 6× Xeon E5-2678 v3 CPU.

**Data configuration**: All cell images were resized to an input dimension of $224\times224$ pixels. To improve the robustness of our model, the image augmentation, consisting of random rotation (maximum degree is 30), random vertical flip (probability is 0.5), random horizontal flip (probability is 0.5), and random affine (can translate the image up to 10% in both the horizontal and vertical directions), was applied to images during training phase All images for training and testing were standardized by mean=[0.485, 0.456, 0.406] and std=[0.229, 0.224, 0.225]. For tumor markers data, we first calculated their distribution and then standardized them according to their mean and std.

**Model configuration**: We adopt ResNet-18^1^ without pre-trained weight as the imagery features extractor. Uniformity in feature dimensions is maintained across the board, with all key features, including the extractor's output, cellular feature embedding, tumor marker feature embedding, and the embeddings utilized in the temporal interaction module (TIM), standardized to a dimensionality of 512. Additionally, our model is designed to be flexible and adaptive, allowing the exact number of cells, tumor markers, and time points to vary, and is not fixed and intrinsically tied to specific patient data and the sampling strategy elucidated in the subsequent sections. All attention operations in this study were implemented with two heads. We utilized the common cosine positional embedding strategy to embed time points.

**Training configuration**: We trained our model using 100 epochs, a batch size of 4, and engaged four workers to facilitate data loading. The model was optimized using the Stochastic Gradient Descent (SGD) algorithm, starting with a learning rate of 5e-4, a weight decay of 0.1, and default momentum settings. A multi-step learning rate decay strategy was employed, where the learning rate was reduced by a factor of 0.1 periodically in the 50-th and 75-th epoch to adapt to the diminishing gradient scenario. The loss function of this study was the Cross-Entropy loss, which is a common choice for binary classification tasks.

**Sampling strategy**: Taking into account parallel data loading, cache utilization, and model robustness, we randomly conducted 80% non-return sampling on tumor cells in time dimension, cell dimension, and tumor markers in time dimension during training. This strategy is equivalent to making dropout at the data level, and can also bring more robust performance to the model, adapting to real-world problems such as time loss and object loss. During the verification or testing phase, all data will be utilized and there will not be any random sampling.

## Text S3. Evaluation metrics in oncological analysis.

In our study, the assessment of the model's performance hinges on a robust evaluation using a combination of statistical and clinical metrics. We have incorporated both the Receiver Operating Characteristic Area Under the Curve (ROC-AUC) for the quantitative evaluation of our model's discriminative capacity. The ROC-AUC metric is pivotal for gauging the model's prowess in distinguishing between clinically defined groups - in this case, potential treatment responders and non-responders. The ROC curve is a graphical representation that contrasts the true positive rate (TPR) against the false positive rate (FPR) for diverse thresholds set for binary classification. The essence of this curve lies in its ability to provide insights into the balance between sensitivity (TPR) and specificity (1-FPR). The Area Under the Curve (AUC) further encapsulates the model's overall performance, providing a singular value for comparative analysis. An AUC value ranging between 0.5 (indicating no predictive ability) to 1.0 (indicative of an impeccable model) serves as a yardstick to determine the model's efficacy in the clinical domain.

References

1. He K, Zhang X, Ren S, Sun J. Deep residual learning for image recognition. Proceedings of the IEEE conference on computer vision and pattern recognition; 2016; 2016. p. 770-8.

2. Dosovitskiy A, Beyer L, Kolesnikov A, et al. An image is worth 16x16 words: Transformers for image recognition at scale. *arXiv preprint arXiv:201011929* 2020.

3. Vaswani A, Shazeer N, Parmar N, et al. Attention is all you need. *Advances in neural information processing systems* 2017; **30**.

4. Paszke A, Gross S, Massa F, et al. Pytorch: An imperative style, high-performance deep learning library. *Advances in neural information processing systems* 2019; **32**.
